# Biologically driven cut-off definition of lymphocyte ratios in metastatic breast cancer and association with exosomal subpopulations and prognosis

**DOI:** 10.1038/s41598-020-63291-2

**Published:** 2020-04-24

**Authors:** Lorenzo Gerratana, Debora Basile, Barbara Toffoletto, Michela Bulfoni, Silvia Zago, Alessandro Magini, Marta Lera, Giacomo Pelizzari, Pietro Parisse, Loredana Casalis, Maria Grazia Vitale, Valentina Fanotto, Marta Bonotto, Federica Caponnetto, Michele Bartoletti, Camilla Lisanti, Alessandro Marco Minisini, Carla Emiliani, Carla Di Loreto, Gianpiero Fasola, Francesco Curcio, Antonio Paolo Beltrami, Daniela Cesselli, Fabio Puglisi

**Affiliations:** 1 Department of Medicine (DAME), University of Udine, Udine, 33100 Italy; 2 Department of Medicine, Division of Hematology and Oncology, Robert H Lurie Comprehensive Cancer Center, Feinberg School of Medicine, Northwestern University, Chicago, IL 60611 USA; 3 Department of Oncology, ASUFC University Hospital, Udine, 33100 Italy; 4 Anatomic Pathology Institute, ASUFC University Hospital, Udine, 33100 Italy; 5 Clinical Pathology Institute, ASUFC University Hospital, Udine, 33100 Italy; 6 Department of Chemistry, Biology and Biotechnology, University of Perugia, Perugia, 06122 Italy; 7 INSTM-ST Unit, Area Science Park, Trieste, 34149 Italy; 8 Elettra-Sincrotrone Trieste S.C.p.A., Area Science Park, Trieste, 34149 Italy; 9 Department of Medical Oncology, Centro di Riferimento Oncologico (CRO), IRCCS, 33081 Aviano, Italy

**Keywords:** Breast cancer, Tumour biomarkers

## Abstract

High neutrophil to lymphocyte ratio (NLR) and monocyte to lymphocyte ratio (MLR) are respectively associated with systemic inflammation and immune suppression and have been associated with a poor outcome. Plasmatic exosomes are extracellular vesicles involved in the intercellular communication system that can exert an immunosuppressive function. Aim of this study was to investigate the interplay between the immune system and circulating exosomes in metastatic breast cancer (MBC). A threshold capable to classify patients according to MLR, NLR and PLR, was computed through a receiving operator curve analysis after propensity score matching with a series of female blood donors. Exosomes were isolated from plasma by ExoQuick solution and characterized by flow-cytometry. NLR, MLR, PLR and exosomal subpopulations potentially involved in the pre-metastatic niche were significantly different in MBC patients with respect to controls. MLR was significantly associated with number of sites at the onset of metastatic disease, while high levels of MLR and NLR were found to be associated with poor prognosis. Furthermore, exosomal subpopulations varied according to NLR, MLR, PLR and both were associated with different breast cancer subtypes and sites of distant involvement. This study highlights the nuanced role of immunity in MBC spread, progression and outcome. Moreover, they suggest potential interaction mechanisms between immunity, MBC and the metastatic niche.

## Introduction

The role played by the immune system in cancer pathophysiology is central^1^. According to the immunoediting theory, it plays indeed a dual function, by both protecting the organism against cancer and, on the other hand, facilitating cancer’s escape and promoting its progression^2^. Accordingly, the patients’ clinical outcome may be influenced not only by the histopathological characteristics of the tumor itself, but also by the host reaction, including inflammatory response. Recent studies have confirmed the role of the host’s inflammatory response in tumor development and progression, including breast cancer (BC); which is the most common form of malignancy among women^3,4^. During the systemic inflammatory response, proportions between neutrophils, lymphocytes, and monocytes may change, and neutrophilia, thrombocytosis, and relative lymphocytopenia could be observed in the peripheral blood^5,6^. Hence, these parameters have been investigated as prognostic markers in different cancers, either as absolute count or as ratios. Of these, neutrophil-to-lymphocyte ratio (NLR) is the most widely evaluated as its elevation is associated with poor prognosis in several cancers^7^. Similarly to NLR, also lymphocyte-to-monocyte ratio (LMR) and platelet-to-lymphocyte ratio (PLR) could reflect the imbalance between adaptive and innate immune system and thus, an inadequate anti-tumor activity^8,9^. Indeed, circulating monocytes could be recruited at tumor site and differentiate into macrophages, where they are further involved in tumor development, dissemination, angiogenesis, matrix degradation, and immunosuppression; whereas lymphopenia could be linked to a weak anti-tumor response and lower tumor-infiltrating lymphocytes^10,11^. Lower LMR has been associated with poor survival in non-hematological and hematological malignancies^8^. As for BC, the results of several studies investigating the relationship between LMR and prognosis are concordant, showing that a low LMR is associated with worse prognosis, though some differences could be observed^12–15^. Likewise, platelets participate to the inflammatory response and moreover interact directly with tumor cells and may contain factors that contribute to tumor growth, invasion, and angiogenesis^16^.

Since a better risk stratification is of pivotal importance in the patients’ clinical management, the implementation of such cost-effective biomarkers could be promising to design new tailored therapeutic approaches^17^.

On the other hand, an important critical aspect of these easy-to-collect hematological indexes, at present, is the lack of a consensus cut-off value to reliably stratify the risk of recurrence and mortality.

Exosomes, extracellular vesicles of endocytic origin characterized by 40–100 nm diameter, are part of the intercellular communication system^18–20^ In fact, they are released by all the tested cells, can be found in all the biological fluids, including plasma, and their molecular content, comprising proteins, lipids and nucleic acids, can be delivered to target cells^19,20^. In this way, the exosome content, strictly related to the nature and the status of the cell of origin, can modulate both phenotype and function of recipient cells^19,20^. For this reason, circulating exosomes are under investigation as accessible biomarkers for cancer detection and monitoring^21^. Indeed, accumulating reports suggest that tumor-derived exosomes may impact tumor growth, angiogenesis, tissue invasion and metastasis^22^ as well as favor immune escape^23^. Similarly, exosomes released by an activated tumor microenvironment can increase tumor aggressiveness and immune-escape^24,25^. In this regard, all Antigen Presenting Cells (APCs) are profoundly affected by tumor derived exosomes (TDE), that not only impair the capacity of circulating CD14 + monocytes to differentiate into functional DCs, but also lower or disable their expression of HLA-DR^24,26^. These altered cells suppress lymphocyte proliferation and impair the expression of effector molecules such as IFN-γ and perforin, similarly to what is observed among natural killer cells^24,27^. Accordingly, we have previously shown that exosomes derived from the plasma of glioblastoma patients were able to reduce the proliferation of T lymphocyte and that this effect was mediated by CD14 + monocytes. Indeed, exosomes were preferentially internalized by monocytes^28^. Therefore, the aim of this study was to evaluate the interplay between MLR, NLR, PLR and exosomal subpopulations in metastatic BC patients to provide real world insights of the mutual interaction between immunity and metastatic niche. Furthermore, the prognostic role of LRs was investigated both singularly and after their combination.

## Materials and Methods

### Study design

The overall study was composed by three distinct cohorts.

The main retrospective cohort (MBC_main) was built by reviewing the medical records of 396 patients receiving anticancer treatments for MBC at the Department of Oncology, University Hospital of Udine, Italy, from January 2006 to January 2017.

A second cohort was composed by 1128 healthy female blood donors and used as control. Consequently, two subgroups were generated through propensity score matching of the MBC_main population with healthy subjects. The matched MBC subgroup was defined as MBC_psm. Complete blood counts were performed by the Department of Laboratory Medicine, University Hospital of Udine.

A third exosome-focused cohort (MBC_exo) comprised 68 MBC patients prospectively enrolled between March 2013 and May 2015, regardless of the line of treatment. Patients’ blood samples underwent exosomes isolation and characterization at the Department of Pathology, University Hospital of Udine.

The study was approved by the Departmental Review Board, Department of Oncology, University Hospital of Udine and by the Ethics Committee (approved November 2017).

An ad-hoc protocol was developed and approved by the Ethics Committee (amendment No. 178/2014/Em) for the prospective cohort. All patients gave their written informed consent before their enrollment and all study procedures were carried out in accordance with GCP guidelines and clinical research regulations.

The datasets generated and analyzed during the current study are not publicly available due to relevant data protection laws. The data may be available upon reasonable request to the corresponding author.

### Statistical analysis

Clinico-pathological characteristics of the MBC_main cohort were summarized through descriptive analysis. Categorical variables were described through frequency distribution, whereas continuous variables were reported through median and interquartile range. MLR, NLR, PLR and exosomal subpopulations distributions were tested for normality by Shapiro–Wilk test and their association with metastatic disease and clinico-pathological features was explored by Wilcoxon rank-sum test or Kruskal–Wallis test, as statistically appropriate.

To identify a threshold capable to classify patients according to MLR, NLR and PLR, a receiving operator curve (ROC) analysis was performed, after propensity score matching (PSM) with a series of 1228 female blood donors. Propensity score was computed by taking into consideration age. The matching approach was 1:1 nearest neighbor with caliber of 20%^29,30^. The optimal threshold was calculated through empirical cut point estimation with Liu method^31^.

Overall survival (OS) was defined as the time between treatment start and death from any cause. Prognostic factors in terms of OS were tested both in uni- and multivariate models by Cox regression with 95% confidence interval (95% C.I.). Differences in survival were tested by log-rank test and represented by Kaplan-Meier estimator plot.

Variations in MLR, NLR and PLR between first and second line were analyzed through Wilcoxon signed-rank test.

Statistical analysis was performed using STATA (StataCorp. (2015) Stata Statistical Software: Release 14.2. College Station, TX: StataCorp LP), R (The R foundation for Statistical Computing. version 3.3.1 (2016-06-21)) and SPSS 23 (IBM Corp. Released 2015. IBM SPSS Statistics for Windows, Version 23.0. Armonk, NY: IBM Corp.).

### Blood sample analysis

NLR and PLR were defined as the absolute neutrophil count divided by the absolute lymphocyte count, and the absolute platelet count divided by the absolute lymphocyte count, respectively; MLR was the result of the ratio between the absolute monocyte count and absolute lymphocyte count. These parameters were analyzed from peripheral blood cell count by DxH 800 hematology analyzer (Beckman Coulter). Full blood count data were eligible for analysis if performed within 2 months before the start of first line and 3 days for second-line.

### Exosome precipitation and characterization

#### Exosome precipitation

Exosome assessment was performed before the beginning of a new therapeutic line. Exosome were precipitated from 1 ml of “platelet-free plasma” by ExoQuick Exosome Precipitation Solution (SBI System Biosciences) according to manufacturer’s protocol^25^. Briefly, fresh serum was centrifuged 30 min at 3000 g at 4 °C. To 250 µl of serum were added 63 µl of ExoQuick and incubated at 4 °C 30 min. After this period, the sample was centrifuged 45 min at 1000 g and the supernatant was eliminated. Again, the pellet was centrifuged 5 min at 1000 g to remove all traces of fluid and exosome pellet was resuspended in 200 μl of Dulbecco’s Phosphate Buffered Saline (PBS). “Platelet-free plasma” samples were obtained from ethylenediamine tetraacetic acid (EDTA)-added peripheral blood samples by two sequential centrifugations (2600 rpm for 15 minutes at room temperature (RT) followed by the centrifugation of the supernatant at 13000 g for 15 minutes at 4 °C). Before exosome precipitation, “platelet-free plasma” was pre-treated with 5U/ml Thrombin (SBI System Biosciences) following manufacturer instructions.

#### Exosome purification by sucrose linear density gradient

In order to demonstrate the presence of exosomes in the ExoQuick enriched fractions, Fresh serum was centrifuged 30 min at 3000 *g* at 4 °C. To 500 µl of serum were added 126 µl of ExoQuick (System Biosciences) and incubated at 4 °C 30 min as reported in the manufacturer’s protocol. After this period, the sample was centrifuged 45 min at 1000 *g* and the supernatant was eliminated. Again, the pellet was centrifuged 5 min at 1000 *g* to remove all traces of fluid and exosome pellet was resuspended in 100 μl of D-PBS. Exosomes were then isolated by floatation in linear sucrose gradient. Briefly, exosomes isolated by Exoquick (100 μl) were resuspended in 400 µl of 20 mM Hepes pH 7.4 containing 2.50 M sucrose. The samples were transferred into polyallomer centrifuge tube, then carefully overlaid (by peristaltic pump) with continuous sucrose gradient (from 2.00 M sucrose in 20 mM Hepes pH 7.4 to 0.25 M sucrose in 20 mM Hepes pH 7.4). Centrifugation was performed for 16 h at 40000 rpm, 4 °C (Optima Max ultracentrifuge and MLS-50 rotor; thinwall polyallomer tube). After ultracentrifugation, ten fractions of 0.5 ml were recovered from top (fraction 1) to bottom (fraction 10). Each gradient fraction was subjected to ultracentrifugation to eliminate sucrose and concentrate exosomes. Fractions (0.5 ml) were diluted with 2.5 ml of 20 mM HEPES, pH 7.4 and centrifuged in 3 ml tubes 1 h at 50000 rpm, 4 °C (Optima Max ultracentrifuge and TLA-100.3 rotor; thickwall polycarbonate tubes). Exosome quantification was performed by Bradford’s assay after lysis of the samples in RIPA lysis buffer (NaCl 150 mM, 1X NP40, 0.1% SDS, 1% sodium deoxycholate, 25 mM Tris HCl pH 7.6, all of Sigma-Aldrich) in the presence of protease inhibitors (ThermoScientific).

#### Atomic force microscopy (AFM) analyses

AFM analysis was carried out adsorbing for 30 minutes 100 μl of exosomes enriched by ExoQuick and diluted in PBS on freshly cleaved 11 × 11 mm mica sheets (Agar Scientific). Imaging was performed in liquid (PBS) on a MFP-3D Stand Alone (Oxford Instruments GmbH, Wiesbaden, Germany) in dynamic mode with silicon probes (Force constant 0.5–1 N/m, radius of curvature <10 nm, NSC36 Mikromasch, Sofia, Bulgaria). Topographic height images were acquired at 256×256 and 512 × 512 pixels with a scan rate of 1–2 Hz. Image processing and data analysis has been performed using Igor Pro and Gwyddion softwares.

#### Nanoparticle tracking analysis (NTA) analyses

Assessment of exosomes by NTA was performed on a NanoSight LM10 system (Malvern) by analyzing ∼500 μl of ExoQuick-enriched exosome preparations properly diluted in PBS (∼10^3^–10^4^ times). Individual videos of 60 seconds for each sample were acquired using the maximum camera gain and analyzed by the NanoSight particle tracking software to determine particles size and density.

#### Western blot analysis

Samples were resuspended in sample buffer 1X and subjected to 10% Sodium Dodecyl Sulphate - PolyAcrylamide Gel Electrophoresis (SDS-PAGE) under reducing or non-reducing conditions, respectively. Proteins were transferred to Polyvinylidene difluoride (PVDF) membrane and reacted with primary antibodies, overnight at 4 °C, at the following dilutions: 1:500 for mouse monoclonal anti-CD9, 1:500 for mouse monoclonal anti-CD63. After being washed, the membranes were incubated with secondary anti-mouse IgG and developed by Enhanced ChemiLuminescence (ECL).

#### Dynamic light scattering (DLS)

Exosomes from pooled CD9-positive fractions 3–5 were subjected to DLS. DLS is a technique for measuring the size and size distribution of molecules and particles dispersed or dissolved in a liquid. The Brownian motion of particles or molecules in suspension causes laser light scattering at different intensities. Analysis of these intensity fluctuations yields the velocity of the Brownian motion and hence the particle size using the Stokes-Einstein relationship.

#### Scanning electron microscope (SEM) analysis

CD9 positive fractions (Fractions 3–6) were thawed from a minus 80 °C freezer and prepared for SEM imaging. Both samples were diluted properly (at least 100 times) in PBS (pH 7.4) and volume of 10 μl of each dilution were deposited on a pure, thin glass substrate. The plates with fixed exosomes were stored in 4 °C temperature for gentle drying. So-prepared exosomes underwent immediate gold/palladium (80:20, 60 seconds) sputtering for scanning electron microscopy visualization. Structure of exosomes was analysed by scanning electron microscopy SEM (Jeol, JSM 7001 F TTLS). The etched surfaces were coated for 60 seconds with gold/palladium (80:20) using sputter coater/turbo evaporator (Quorum Technologies Q150T ES) in order to provide an electrically conductive thin film to reduce thermal damage and charging of the samples. The SEM micrographs were acquired by applying the accelerating voltage of 15 kV and SEI secondary electron methodology.

### Cytofluorimetric analysis of exosomes

Because of the resolution limit of a flow-cytometer, exosomes were bound to 4-μm aldehyde/sulfate latex beads (Molecular Probes, Invitrogen).

Briefly, latex beads were firstly functionalized with anti-CD63 antibodies by mixing and incubating overnight at 4 °C equal amounts of 0.5 mg/ml anti-CD63 antibodies (BD Biosciences) and 20 mg/ml latex beads. After blocking of the reaction by 0.1% glycine (Sigma-Aldrich), latex beads are re-suspended in PBS/0.1% BSA at the final concentration of 300,000 beads/μl. Exosomes are bound to latex beads by incubating 10 μg of exosomal proteins, resuspended in 100 μl of 1x PBS, with 2 μl of CD63-coated beads, corresponding to 200000 CD63-coated beads, for 15 at room temperature. Reactions were stopped by 0.1% glycine in PBS and, after washes with 0.05% PBS/Tween, incubated with 2 μl of anti-Fc receptor (Miltenyi Biotec).

Finally, exosomes bound to latex beads were incubated with the following primary antibodies: CD9 PE, KDR APC (BD Biosciences); EGFR Alexa 488, CD44 APC.Cy7, EpCam FITC, Her2 PE, CD49d APC (BioLegend); CXCR4 FITC, c-MET APC (R&D Systems); CD9 APC (Serotec); CD63 FITC (Santa Cruz Biotechnology). Controls were obtained by staining with properly labelled isotypic controls. Samples were acquired with FACSCanto II (BD Biosciences), while the analysis was carried out through the Summit program (Dako Cytomation). Specifically, it was considered as exosomes the population of events characterized by a size of 4-μm and positivity for CD63 and/or CD9).

### *In vitro* proliferation of T cells after co-culture with exosomes

The immunomodulatory effect of exosomes on CD3 + T cells were assessed as previously described^1^. Briefly, 2 × 10^5^ peripheral blood mononuclear cells (PBMCs) were resuspended in 200 μl of medium and incubated for 24 hours with 1.5 ×1010 plasma derived exosomes precipitated by either healthy controls (n = 2) or MBC patients (n = 2). PBMCs were labelled with 5 μM carboxyfluorescein succinimidyl ester (CFSE, Invitrogen) in PBS with 0.1% bovine serum albumin for 10 minutes at 37 °C, followed by immediate quenching with cold culture medium and then seeded into 96 wells with pre-bound 0.5 μg/ml anti-CD3 (clone OKT3, eBiosciences) and 0.5 μg/ml anti-CD28 (clone CD28.6, eBiosciences) and stimulated with different exosome dilutions (1:2, 1:100), with respect to the initial volume of plasma sample). Negative controls (unstimulated PBMCs) and positive controls (stimulated PBMCs without exosomes) were also assayed. After 3 days, PBMCs were stained with anti-CD3 and tested by flow cytometry for CFSE staining (FACSCanto II, Becton Dickinson).

### Exosome internalization experiments

To compare the ability of CD14 + monocytes and CD3 + lymphocytes to internalize plasma-derived exosomes, 1.5 × 10^10^ exosomes plasma-derived were resuspended in PBS and stained with 5 μM DiD for 30 minutes at 37 °C. 2 × 10^5^ isolated PBMCs were then incubated with DiD-labelled vesicles for 5 and 24 hours, respectively, and subsequently stained for CD45, CD3 and CD14 (Becton Dickinson) and analyzed by flow cytometry (FACSCanto II, Becton Dickinson) by gating either on CD14 + or CD3 + PBMCs.

## Results

### LRs are significantly different with respect to healthy controls

This retrospective study analyzed a consecutive cohort of 396 patients with a diagnosis of MBC between January 2006 and 2017 (MBC_main patients) and 1128 blood donors. Among the analyzed patients, 42 were younger than 45 years, 186 were aged between 45 and 65 years, and 168 were older than 65 years. Median follow-up was 53 months, and the median overall survival was 31 months. Survival was 78.56% at 12 months, 55.98% at 24 months, and 27.49% at 60 months. Patients’ clinico-pathological characteristics are described in Table 1.

**Table 1:** Patients’ characteristics.

	N	%
**Age (N** = **396)**		
≥45 and ≤65	186	(47.0)
<45	42	(10.6)
>65	168	(42.4)
**Grading (N** = **324)**		
1	11	3.4
2	162	0.50
3	151	46.6
**Histotype (N** = **390)**		
Ductal	318	(81.6)
Lobular	59	(15.1)
Others	13	(3.3)
**ER status (N** = **334)**		
Negative	80	(24.0)
Positve	254	(76.0)
**PR status (N** = **328)**		
Negative	126	(38.4)
Positve	202	(61.6)
**Ki67 (N** = **334)**		
<14%	70	(21.8)
≥14%	251	(78.2)
**HER2 (N** = **334)**		
Uncertain	2	(0.6)
Negative	245	(73.4)
Positive	87	(26.0)
**Profiles (N** = **310)**		
Luminal A	44	(14.2)
Luminal B	151	(48.7)
Luminal HER2	43	(13.9)
HER2 Positive	35	(11.3)
Triple Negative	37	(11.9)
**ECOG Performance Status (N** = **389)**		
0	204	(52.4)
1	150	(38.6)
2	35	(9.0)
**Number of Sites (N** = **396)**		
1	213	(53.8)
2	105	(26.5)
3	53	(13.4)
4	19	(4.8)
5	5	(1.3)
6	1	(0.2)

NLR, MLR and PLR were significantly different in MBC_main patients with respect to blood donors (p = 0.0001 for MLR, NLR and PLR). On the other hand, no significant differences were observed according to the type of metastatic onset (i.e. *de novo* versus relapsed).

In order to calculate a threshold for NLR, MLR, PLR capable to stratify patients’ population, a ROC analysis was performed on a propensity score matched pooled cohort comprising both blood donors and MBC patients. The optimal cut-off identified for MLR was 0.28 (AUC = 0.6447), 2 for NLR (AUC = 0.7439), and 148.38 (AUC = 0.6766) for PLR. Patients with LRs equal or above these thresholds were defined as MLR^high^, NLR^high^ and PLR^high^, respectively.

### Exosomal subpopulations are significantly different with respect to healthy controls

**Figure 1:**
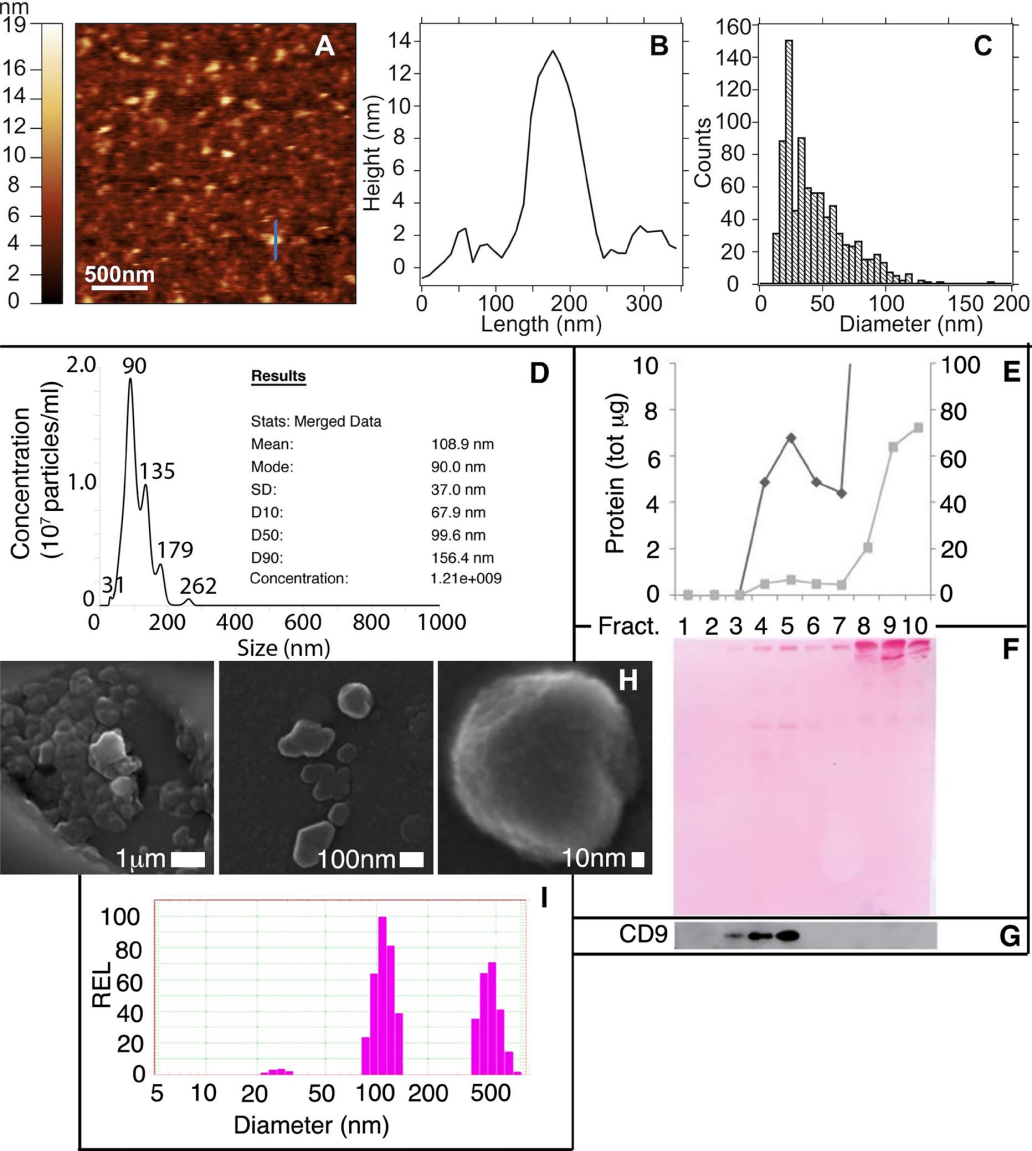
Characterization of Exoquick-precipitated plasma exosomes. (**A–C**) Atomic Force Microscopy (AFM). (**A)** Representative AFM micrograph (2.5 μm × 2.5 μm) of mBC exosomes enriched by ExoQuick, diluted in PBS and deposited on a mica substrate. **(B)** Height profile of a single vesicle (blue line in panel A) matching the typical lateral dimensions of exosomes (<150 nm). **(C)** Quantification of the exosome diameter: histogram show the distribution of the diameter of exosomes, as extracted by grain analysis (Gwyddion) of three distinct AFM images of the same sample. **(D)** Nanoparticle Tracking Analysis (NTA). Representative histogram showing the particle size distribution of an exosome preparation analyzed by Nanosight and diluted 1:1000 in water. **(E–G)** Western Blot. (**E)** After ExoQuick precipitation, samples were subjected to floatation on a linear sucrose gradient. Protein distribution in the gradient is shown as µg of proteins recovered in each fraction (grey line; scale on the right). Enlarged data (black line; scale on the left) are also shown. **(F)** Ponceau staining of the gel. **(G)** Cropped immunoblot showing CD9 immunoreactivity of the different recovered fractions (full length blot is displayed in Supplementary Fig. 1). **(H)** Scanning Electron Microscopy (SEM). Representative pictures, acquired at different magnification, of a sample whose CD9 positive fractions (fractions 3–5) were pooled and subjected to SEM analysis. **(I)** Dynamic light scattering (DLS) analysis. CD9 positive fractions 3–5 were pooled and analyzed by DLS. Histograms represent the size distribution of the vesicles. The profile shows the presence of distinct populations. The first one with an average size of about 30 nm and the second one of 110 nm. A population with an average diameter of 450 nm was also observable, probably due to protein aggregation induced by ExoQuick treatment.

Plasma exosomes were precipitated by using ExoQuick, a commercial kit based on the use of polyethylene glycol (PEG). Atomic Force Microscopy (Fig. 1A–C) and Nanoparticle Tracking Analysis (NTA) by Nanosight (Fig. 1D) confirmed the presence, in precipitated preparations, of vesicles with a diameter compatible with that of exosomes (10^th^, 50^th^ and 90^th^ percentiles: 67.4 nm, 99.1 nm and 155.8 nm, respectively; n = 10), with an average concentration of the order of 10^12^ particles per ml of plasma. To verify that the isolated particles were enriched in exosomal markers we first analyzed, by western blot, the expression of markers such as CD9, Alix and enolase. Since Exoquick is able to precipitate also the proteins of the plasma, diluting the amount of exosomal proteins, Exoquick-enriched preparations were further subjected to floatation on linear sucrose density gradient (2–0.25 M; Fig. 1E,F). Of the 10 fractions collected it was shown that fractions 3 to 5 of the gradient were enriched in CD9 (Fig. 1G). The same fractions analyzed by SEM and DLS showed the presence of particles whose morphology and size were compatible with exosomal particles (Fig. 1H,I).

**Figure 2:**
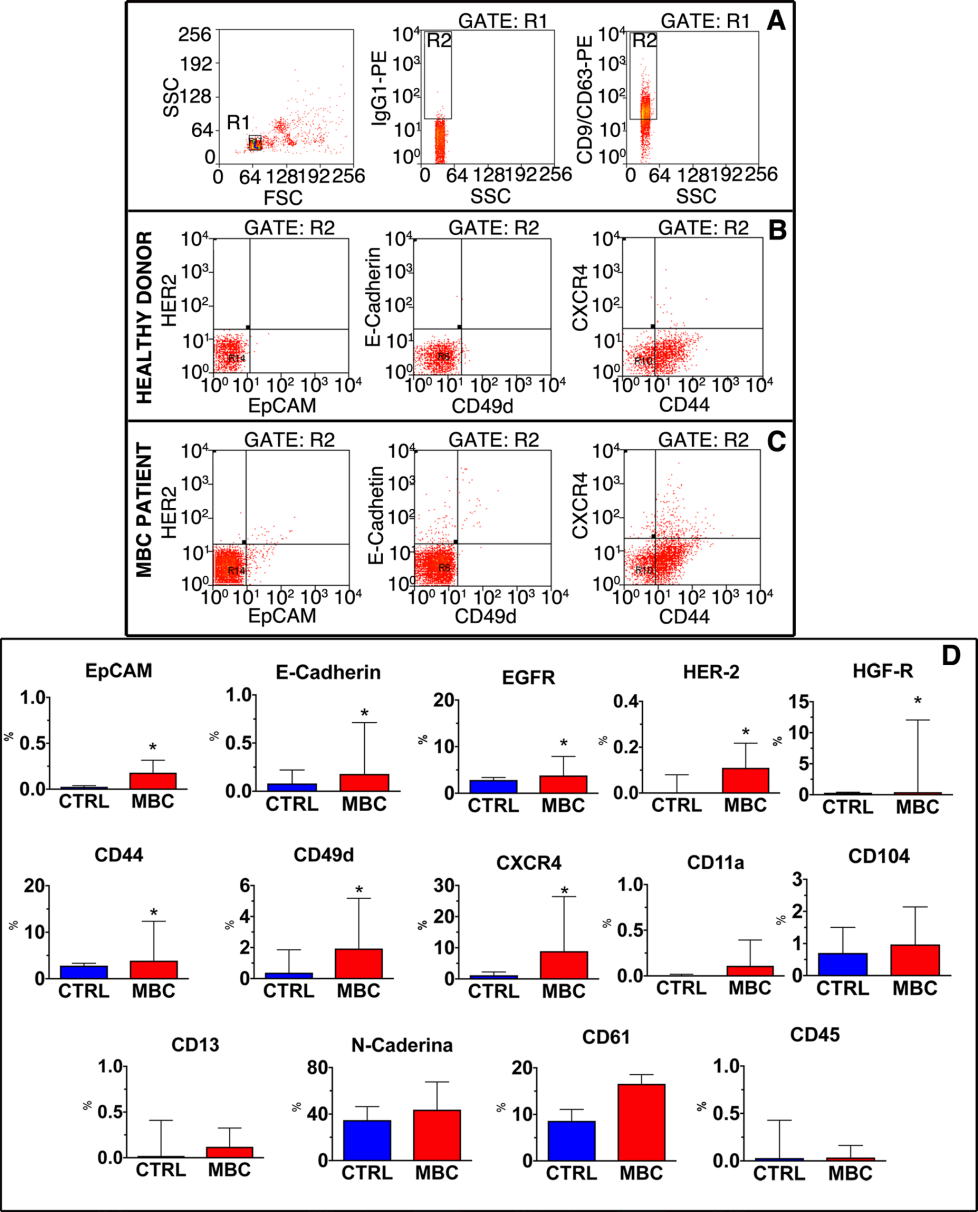
Characterization of exosomes by FACS. (**A**) Gating strategy. Taking advantage of beads of specific size, the gate corresponding to latex beads (gate R1) was identified in the FSC/SSC dot plot (left panel). Events included in R1 were evaluated for the positivity to the isotypic specific antibody to define the gate of positivity for the exosome specific markers CD9 and CD63 (central panel). Exosome-positive beads were therefore included in the R2 gate (right panel). **(B,C)** Exosome characterization. Representative dot plots defining the fraction of exosome-positive beads expressing HER2, Epcam (left panels), E-Cadherin, CD49d (central panels), CXCR4 and CD44 (right panels) in healthy donors **(B)** and MBC patients **(C)**. **(D)** Quantitative analysis. Data, obtained from the analysis of 20 healthy donors (CTRL) and 20 MBC patients (MBC), are presented as Box and whisker plot. *p < 0.05 vs. CTRL.

Once demonstrated that ExoQuick preparations were indeed enriched in exosomes, we proceeded with a more extensive phenotypic characterization of exosomes adopting a cytofluorimetric protocol meant to detect and characterize exosomal subpopulations on a multiparametric basis. Since exosomes are smaller than the cytofluorimeter resolution limit, 4μm latex beads functionalized with anti-CD63 antibodies were used to capture extracellular vesicles. The presence of these latter was subsequently confirmed by the positivity for CD9 and CD63, and CD9/CD63 positive subpopulations were finally analyzed for the expression of different markers, i.e. the fraction of exosomes expressing markers of epithelial origin (EpCAM, E-Cadherin, EGFR and HER2), markers of exosomes possibly involved in the metastatic process (HGF-R, CD44, CD49d, N-Cadherin, CXCR4), immune-related markers (CD45, CD13, CD11a, CD104), and platelet-derived (CD61) and endothelial-derived (CD31) exosomes (Fig. 2A–C). Comparing 20 healthy donors with 20 MBC patients, differences in the relative composition of plasma exosomes were detected, as displayed in Fig. 2D. Specifically, the fraction of exosomes expressing epithelial markers, such as EpCAM, HER2 and E-Cadherin, was very low in donors and increased, although representing a small fraction of exosomes, in MBC patients, reaching a statistical significance for EpCAM and E-Cadherin. The expression of markers potentially involved in the pre-metastatic niche, such as HGF-R, CD44 and CXCR4 was significantly higher in patients than in controls. These markers were expressed in a fraction of exosomes that reached 10% in patients. No significant differences in the fraction of N-Cadherin, CD61 and markers linked to the immune system were detected among MBC patients and donors. For these reasons, the MBC_exo cohort was assessed for exosomes expressing epithelial and metastatic markers.

### MBC-derived exosomes inhibit T cell proliferation and are preferentially internalized by circulating monocytes

**Figure 3:**
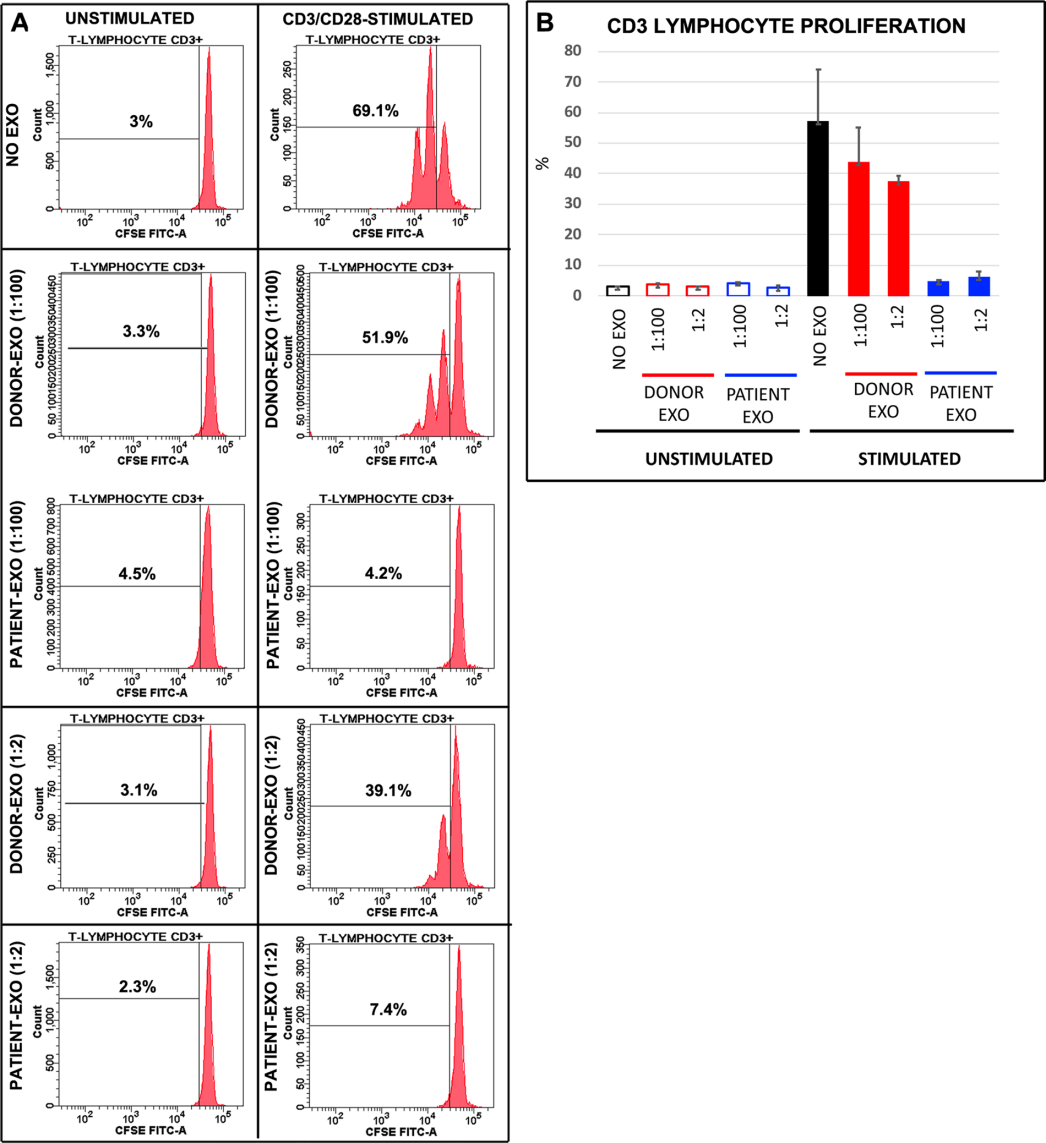
Plasma exosomes derived from MBC patient, but not from healthy donors, inhibit T-cell proliferation. (**A**) CFSE-labeled PBMCs isolated from healthy donors were pretreated for 24 hours without or with plasma-derived exosomes and stimulated or not for 72 hours with anti-CD3 and anti-CD28. The representative CFSE histograms show the fraction of proliferative CD3 + T cells in unstimulated PBMCs (left panels) and stimulated PBMCs (right panels), treated or not with donor-derived or patient derived exosomes, at two different concentrations (1:2 and 1:100). **(B)** Quantification data deriving from two experiments in triplicate are shown. Data are presented as mean ± standard deviation.

**Figure 4:**
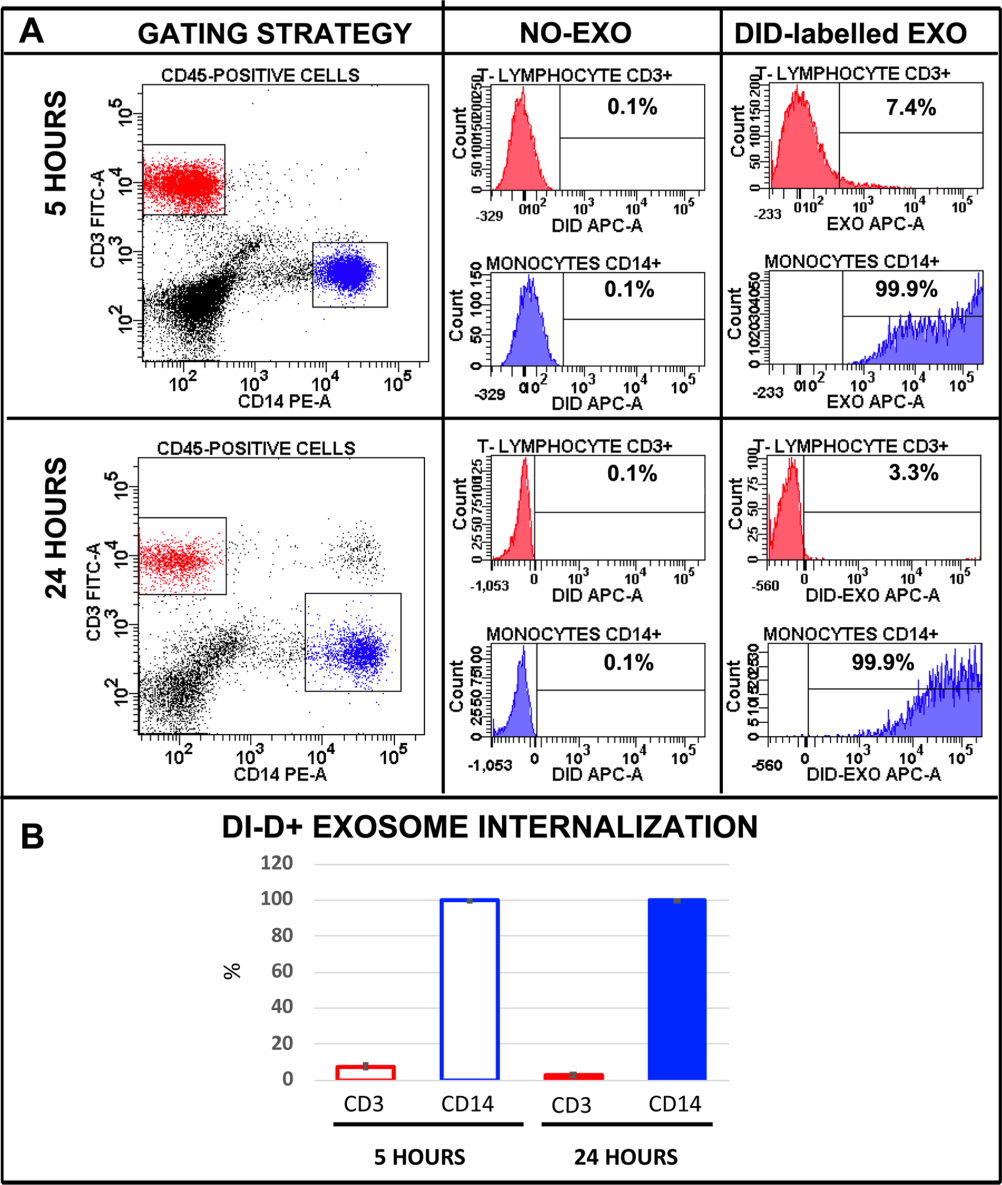
Plasma exosomes are preferentially internalized by monocytes. (**A**) DiD-labelled exosomes were incubated with PBMCs for 5 hours (upper panels) or 24 hours (lower panels) and the uptake by CD14 + monocytes, and CD3 + T cells was measured by flow cytometric analysis. In the dot plots on the left, it is represented the gating strategy to recognize, in the bulk population, T lymphocytes (red dots) and CD14 + monocytes (blue dots). Gated sub-populations were analyzed for DiD-fluorescence. **(B)** Quantification data deriving from two experiments in triplicate are shown. Data are presented as mean ± standard deviation.

We have previously shown that in glioblastoma patients, plasma-derived exosomes were able to reduce the proliferation of T lymphocyte and that this effect was mediated by CD14 + monocytes^28^. To establish whether also exosomes derived from the plasma of MBC patients were able to reduce the proliferation of T lymphocyte, CFSE-labeled PBMCs, isolated from healthy donors, were pretreated for 24 hours without or with plasma-derived exosomes and stimulated or not for 72 hours with anti-CD3 and anti-CD28. As shown in Fig. 3, the proliferation of T-lymphocytes was abolished by MBC-derived exosomes at both the concentration used (1:2 and 1:100). Conversely, exosomes isolated from the plasma of healthy donors did not affect T cell proliferation (Fig. 3). Additionally, by incubating PBMC with DiD labeled exosomes for 5 or 24 hours, it has been shown that, at both time points, 99.9% of CD14 + monocytes resulted to be DiD-positive, while only a minor fraction of CD3 + T lymphocytes internalized exosomes, independently form the time point (Fig. 4).

Therefore, like in glioblastoma patients, exosomes derived from MBC patients were mainly up-taken by monocytes and suppressed T-cell proliferation, thus posing the rationale for an overall analysis focused on both exosomes and NLR/MLR.

### Exosomal subpopulations vary according to LRs and are both associated with different breast cancer subtypes

The stratification of MBC_main according to BC subtypes, highlighted a significantly higher NLR among patients affected by non-luminal HER2-positive and triple negative disease (p = 0.0019). No statistically significant differences were highlighted for MLR or PLR (Fig. S1). In MBC_exo, MLR^high^ and PLR^high^ patients with HER2 BC had a significant higher fraction of CD49d^pos^ exosomes (P = 0.0086, P = 0.0166 respectively) and a trend for CXCR4^pos^. Patients in the NLR^low^ subgroup showed higher fraction of EPCAM^pos^ (P = 0.0344) when affected by HER2 positive disease and CXCR4^pos^ for ER positive disease (P = 0.0344).

MLR^high^ patients of the MBC_exo cohort had a higher fraction of HGFR^pos^ exosomes (P = 0.0055), while PLR^high^ patients had a higher fraction of HER2^pos^ and EpCAM^pos^ exosomes (P = 0.04). Lymphocyte count higher than 1.8 × 10^3^ μL was associated with low HER2^pos^ and high EpCAM^pos^ levels (P = 0.0082 and P = 0.0193).

### LRs are associated with tumor burden and to different exosomal subpopulations according to sites of distant involvement

When considering the association between MLR, NLR, PLR and number of metastatic sites in the MBC_main cohort, only MLR was significantly associated with number of sites at the onset of metastatic disease (p = 0.0096); on the other hand, NLR was associated with an increase in number of sites (p = 0.036). PLR was associated neither with tumor burden, nor with their increase. Notably, no direct associations were observed between LRs and specific metastatic sites.

Consistently with the hypothesis that exosomes could influence the metastatic niche, we observed that patients in with visceral metastases in the MBC_exo cohort had a higher fraction of vascular endothelial growth factor receptor 2^pos^ (VEGFR-2) (P = 0.0323) and marginally of E-caderin^pos^ exosomes, while lung metastases were marginally associated with higher expression of VEGFR-2^pos^ exosomes.

**Figure 5:**
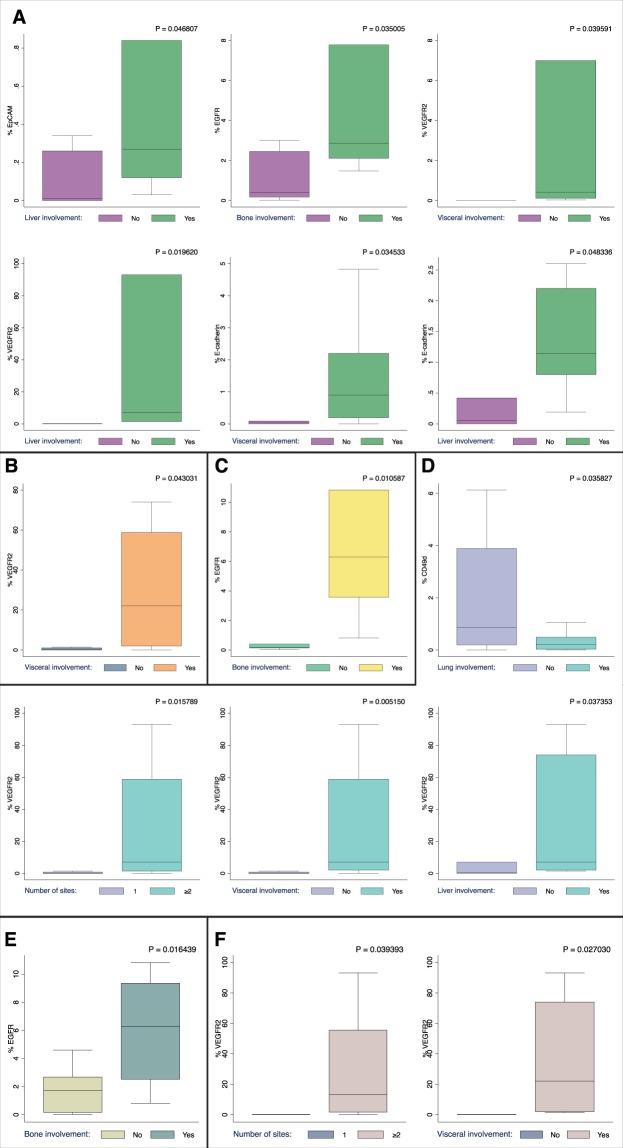
Exosomal subpopulation distribution according to LRs, site of metastasis and tumor burden. (**A**) MLR^low^ patients **(B)** MLR^high^ patients **(C)** NLR^low^ patients **(D)** NLR^high^ patients **(E)** PLR^low^ patients **(F)** PLR^high^ patients. Significant associations are presented as box and whisker plot.

For exploratory purpose, MBC_exo was stratified according to lymphocyte ratios in order to highlight differences according to the immune and inflammatory status. Patients with bone localizations had a higher proportion of EGFR^pos^ exosomes in the MLR^low^, NLR^low^ and PLR^low^ subgroups (P = 0.035, P = 0.01 and P = 0.016 respectively). Notably, patients in the NLR^high^ and PLR^high^ subgroup had high levels of VEGFR^pos^ exosomes when high tumor burden (i.e ≥ 2 metastatic sites) or visceral localizations were detected (NLR^high^ P = 0.0158 and P = 0.0052; PLR^high^ P = 0.039 and 0.027, respectively). Furthermore, patients in the NLR^high^ subgroup had a lower fraction of circulating CD49d exosomes if lung involvement was detected (P = 0.036) and higher VEGFR2 in case of liver localizations (P = 0.037) (Fig. 5).

### LRs impact on prognosis and its magnitude varies across BC subtypes

**Figure 6:**
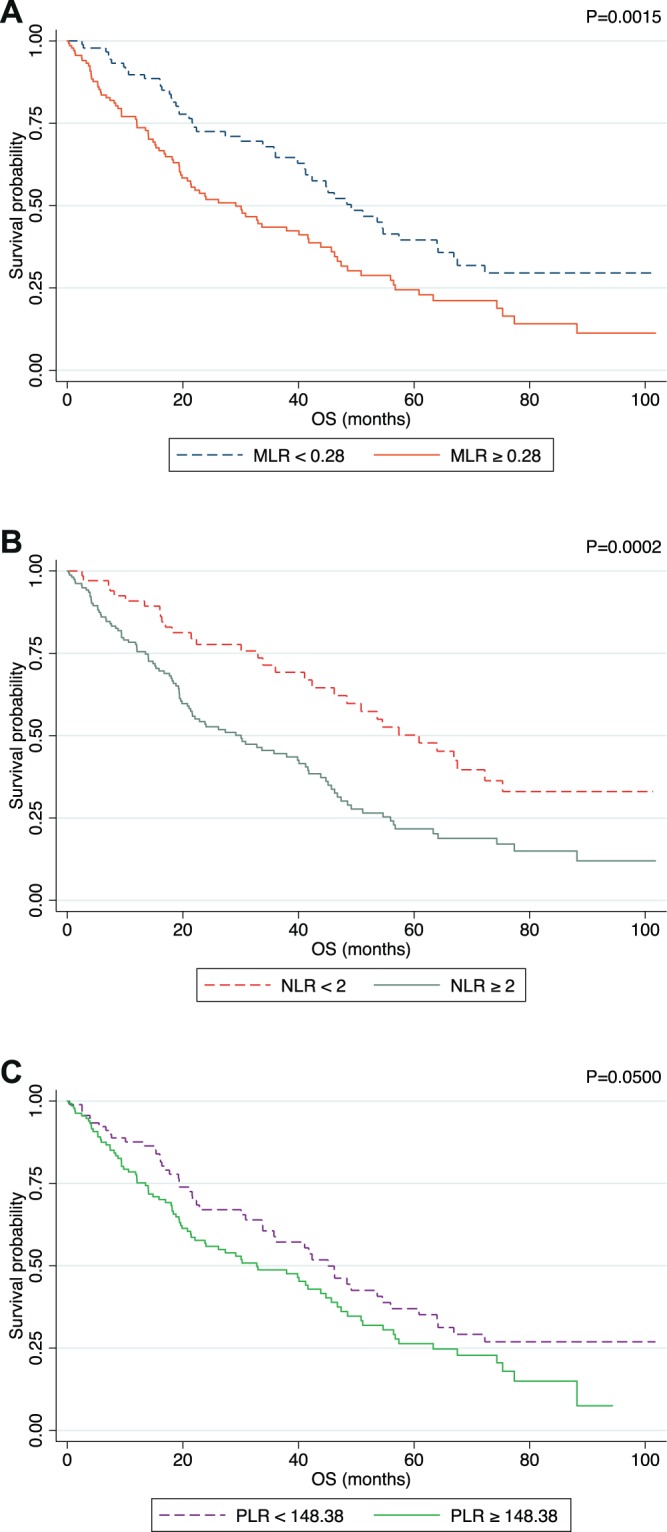
Kaplan Meier plot in terms of OS after applying the ROC Analysis defined threshold for MLR (**A**), NLR **(B**) and PLR (**C**). P value was calculated though log-rank test.

On univariate analysis, MLR^high^ and NLR^high^ patients in the MBC_psm cohort experienced poor prognosis (HR 1.77, 95% CI: 1.24–2.54, p = 0.002; HR 2.09, 95% CI: 1.40–3.12, p < 0.001, respectively). Similar results were observed also in the MBC_main population (data not shown). On the other hand, no significant prognostic impact was observed for PLR (Table 2) (Fig. 6).

**Table 2:** Univariate Cox regression analysis on the matched population.

	HR	95 (%) CI	p value
**Profiles**			
Luminal A	1.00		
Luminal B	1.55	0.84–2.86	0.161
Luminal HER2	1.04	0.49–2.19	0.921
HER2 Positive	1.28	0.57–2.87	0.542
Triple Negative	5.67	2.81–11.43	0.000
**Age**			
≥ 45 and ≤ 65	1.00		
<45	0.81	0.51–1.28	0.375
> 65	0.32	0.08–1.31	0.116
**Metastatic onset**			
Relapsed	1.00		
De novo	0.84	0.59–1.20	0.349
**ECOG PS**			
0	1.00		
1	1.70	1.15–2.50	0.008
2	1.75	1.02–3.01	0.043
**Number of Sites**			
1	1.00		
2	1.61	1.08–2.40	0.019
3	2.13	1.35–3.36	0.001
**Bone only involvement at first line**			
No	1.00		
Yes	0.67	0.42–1.06	0.084
**CNS involvement at first line**			
No	1.00		
Yes	2.11	1.11–4.04	0.023
**Lung involvement at first line**			
No	1.00		
Yes	1.27	0.87–1.85	0.211
**Liver involvement at first line**			
No	1.00		
Yes	1.26	0.87–1.83	0.214
**MLR**			
<0.28	1.00		
≥0.28	1.77	1.24–2.54	0.002
**NLR**			
<1.95	1.00		
≥1.95	2.09	1.40–3.12	0.000
**PLR**			
<149.68	1.00		
≥149.68	1.42	1.00–2.03	0.051
**NLR-MLR Score**			
Score 0	1.00		
Score 1	2.35	1.38–4.02	0.002
Score 2	2.54	1.59–4.07	0.000

Based on these data, MLR and NLR were combined in a score in order to explore the interplay of inflammation and immunocompetence on prognosis. The score ranged from 0 (both MLR and NLR under the cut-off) through 1 (only one between MLR and NLR above the cut-off) to 2 (both ratios above the cut-off). On univariate analysis, the score was capable to stratify the MBC_psm population in 3 groups according to prognosis (OS) (score 1 vs 0 HR 2.35 95% CI: 1.38–4.02, p = 0.002, score 2 vs 0 HR 2.54 95% CI: 1.59–4.07, p < 0.001) (Fig. S2). These results were confirmed in the MBC_main population (data not shown).

Multivariate analysis of the MBC_psm subgroup, showed that only MLR (HR 1.85 95% CI = 1.21–2.83, p = 0.005) and NLR-MLR score 2 vs 0 (HR 1.90 95% CI 1.11–3.26, p = 0.020) retained a significant prognostic impact on OS (Table 3). When applying the models to MBC_main, neither MLR nor NLR nor the NLR-MLR score were independently associated with prognosis (Table 3).

**Table 3:** Multivariare Cox regression models for MLR, NLR and NLR-MLR Score.

	HR	95% CI	P value
Matched population*			
**MLR**			
<0.28	1.00		
≥0.28	1.85	1.20–2.83	0.004
**NLR**			
<2	1.00		
≥2	1.37	0.85–2.19	0.196
**NLR-MLR Score**			
Score 0	1.00		
Score 1	1.38	0.75–2.56	0.300
Score 2	1.90	1.11–3.26	0.19
Total population**			
**MLR**			
<0.28	1.00		
≥0.28	1.37	0.99–1.89	0.053
**NLR**			
<2	1.00		
≥2	1.14	0.80–1.63	0.454
**NLR-MLR Score**			
Score 0	1.00		
Score 1	1.18	0.74–1.88	0.494
Score 2	1.38	0.92–2.08	0.123
HER2-positive and Triple Negative subgroups*			
**MLR**			
<0.28	1.00		
≥0.28	1.92	1.15–3.22	0.013
**NLR**			
<2	1.00		
≥2	0.91	0.48–1.72	0.768
**NLR-MLR Score**			
Score 0	1.00		
Score 1	0.90	0.39–2.08	0.810
Score 2	1.46	0.74–2.89	0.274

^*^Corrected for molecular profiles, ECOG PS, number of sites, CNS involvement at fist line.

**Corrected for molecular profiles, ECOG PS, number of sites, CNS and liver involvement at fist line.

**Figure 7:**
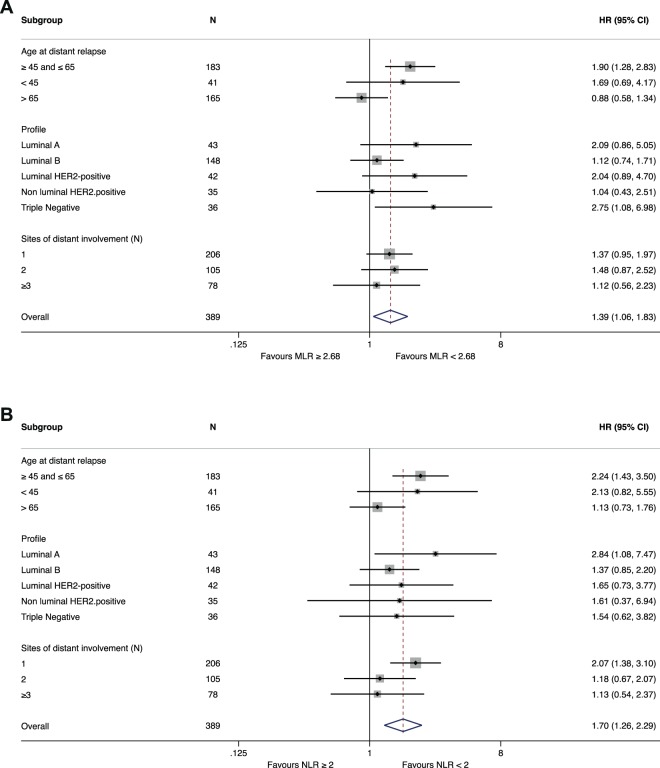
Different prognostic impact of MLR (**A**) and NLR (**B**) according to clinically relevant subgroups. Forest plot highlights a tangible difference in age-defined subgroups, p for interaction = 0.028 and 0.088 for MLR and NLR respectively.

A subgroup analysis was then performed on the MBC_main population to investigate the different impact of lymphocytes ratios according to clinico-pathological features. Test for heterogeneity highlighted a significant interaction according to age for MLR (P for interaction = 0.028) and a trend for NLR (P for interaction = 0.088) (Fig. 7).

Based on the evidence of a greater impact of immunity on triple negative and HER2-positive MBC (luminal and non-luminal), Cox regression was restricted to these subgroups in the MBC_main cohort. On multivariate analysis, only MLR was independently associated with OS (HR 1.92 95% CI: 1.15–3.22, p = 0.013) (Table 3).

**Figure 8:**
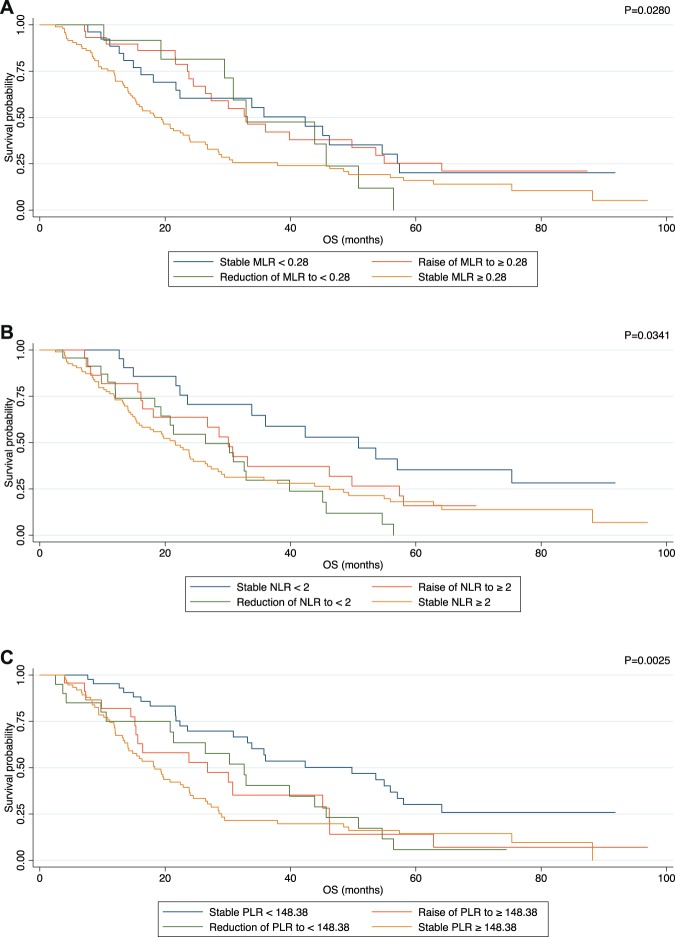
Kaplan Meier plot in terms of OS according to variation of MLR (**A**), NLR (**B**) and PLR (**C**) from the first to the second line of treatment.

The prognostic impact of variation in MLR, NLR and PLR from the first to the second line of therapy was also investigated. Patients of MBC_main with decreased or stable ratios, experienced a better outcome for all the three parameters taken into consideration (i.e. MLR p = 0.028, NLR p = 0.034 and PLR p = 0.003) (Fig. 8).

## Discussion

The present study examined a cohort of 396 patients affected by MBC and investigated the association between NLR, MLR, PLR and exosomal subpopulations. Moreover, the role of LRs on prognosis was explored and its magnitude was related to BC subtypes. LRs have been intensively studied in the last few years in several cancer types, including breast cancer, and the results observed in our study are consistent with those found in literature.

Notably, previous studies based their cut-offs according to literature or on the analyzed population’s prognosis or division in quartiles. The present study adopted an alternative approach using a pooled database composed by propensity score matched blood donors and MBC patients. This strategy highlighted significantly higher values of NLR, MLR and PLR in MBC patients compared to healthy subjects and enabled us to calculate a cut-off on a biological basis and not related to the intrinsic characteristics of the analyzed population. The obtained cut-offs for NLR and PLR are slightly lower with respect to those found in literature (NLR: 1.9; literature range: 2.0 to 5.0, PLR: 150; literature range: 160–300^7,9,32^). Few studies, to date, have investigated the role of monocytes-based parameters in MBC.

This study, moreover, suggests some important aspects linked to the interaction between cancer cells, immunity and the tumor micro-environment. In fact, as previously shown in glioblastoma patients^28^, exosomes isolated from the plasma of MBC patients exerted an immunosuppressive function on T-lymphocytes and were preferentially internalized by monocytes. In a murine model it has been shown that breast cancer-derived exosomes alter macrophage polarization possibly inducing in monocytes a skewing to a cancer-promoting phenotype^33^. Intriguingly, exosomal subpopulations linked to immunity (i.e. CD49d and CXCR4) vary significantly according to HER2, ER status and MLR, NLR and PLR. This result reinforces the notion of a possible connection between specific exosomal subpopulations and immune system. Beside their role in immunity, exosomes are considered to play a significant role in the metastatic spread of a primary tumor, both by participating in oncogenic reprogramming of malignant cells and by contributing to formation of a pre-metastatic niche, thus favoring exosome-mediated organ-specific metastasis^34,35^. Accordingly, results concerning the association of distinct exosomal subpopulations with distant cancer dissemination underline the strict interplay between the immune system and the metastatic niche. The observed reduced frequency of CD49d exosomes, in NLR^high^ patients with lung localizations, could be a consequence of the clustering activity of this integrin during interstitial lung inflammation and secondary to the pivotal role played by neutrophils in the formation of the lung pre-metastatic niche^36,37^. Indeed, *in vitro* studies have shown that CD49d can bind to fibronectin and facilitate C5a-induced neutrophil migration across lung fibroblast monolayers^38,39^. Neutrophils consequently alter the endothelial permeability and the extracellular matrix through lysine oxidase induced collagen cross-linking and matrix metalloproteinases collagen-disruption, potentially favoring circulating tumor cells seeding^36,37^.

Patients with visceral metastasis and high tumor burden (i.e ≥2 metastatic sites) showed increased levels of VEGFR2^pos^ exosomes, suggesting the importance of vascular integrity and neoangiogenesis for the metastatic spread^40^. Interestingly, patients with bone metastasis showed a higher fraction of EGFR^pos^ exosomes in the MLR^low^, NLR^low^ and PLR^low^ subgroups, suggesting a potentially different homing mechanism with respect to visceral involvement. Indeed, it has been pre-clinically demonstrated, that EGFR ligands (e.g. EGF, TGF-β, and amphiregulin) are able to directly stimulate osteolysis when expressed in the bone microenvironment^41,42^. Furthermore, EGF and TGF-β mediated osteoclastogenesis is accompanied by a sustained production of RANKL by bone stromal cells, potentially favoring breast cancer’s bone tropism^43,44^. Notably, patients with visceral localizations were found to have higher EPCAM^pos^ and ECAD^pos^, exosomes consistent with the evidence that surface adhesion molecules displayed on tumor-derived exosomes may influence the metastatization pattern^36,45,46^.

Moreover, elevated MLR and NLR were associated with a worse prognosis, and their combination was capable to further stratify the population in three different prognostic groups^32^. PLR, on the contrary, was found not significantly associated with OS, in accordance to a previous large meta-analysis, but in contrast with the work by Zhu *et al*., which reported a negative prognostic impact on breast cancer patients with a high PLR in terms of OS^9,47^.

The obtained cut-offs are consistently significant when analyzed in the matched population, but some exceptions were found when applied to the general population.

One major reason could be represented by fact that donors older than 65 years old were only 7, and since lymphocyte ratios are deeply affected by the subject’s age, this could have calibrated the thresholds on a younger population of patients. This hypothesis is consistent with the subgroup analysis that highlighted a significant P for interaction for MLR. Furthermore, the subgroup of patients above 65 years is more prone to develop comorbidities and competing risk factors that could hinder a lean evaluation of survival.

Even though several pieces of evidence have shown the pivotal role played by the immune system in triple negative and HER2 positive BC, data concerning lymphocyte ratios in this setting are still widely debated^1,48^.

A recent meta-analysis conducted by Xing *et al*., showed that NLR was found to be associated with OS in HER2 positive and TNBC subtypes, whereas no association was found in luminal A and luminal B tumors^49^. Ethier and colleagues, however, found that the negative prognostic effect of NLR on OS was consistent in all clinico-pathological groups^32^.

This finding has been observed in our cohort when NLR levels were compared between molecular subgroups. Moreover, such differences could explain why MLR retained its significance when the multivariate analysis was restricted to triple negative and HER2-positive breast cancer patients (HR 1.92, 95% CI: 1.15–3.22, p = 0.013).

Jia *et al*., showed that NLR is stronger than MLR in predicting OS in all BC subtypes, as opposed to our results. However, both NLR and MLR, consistently failed to retain their significance among patients affected by luminal-like breast cancer^50^. Conversely Ji *et al*., found LMR to be associated with survival in luminal BC patients^13^. The weak prognostic impact of NLR observed in our cohort could be due to the high proportion of patients with luminal-like disease. In these cases, NLR was remarkably lower, with values often close to the cut-off.

Since the interaction between immunity and tumor cells is dynamic, analyzing of the impact of lymphocyte ratio’s fluctuations might be crucial.

Previous studies assessed NLR, MLR and PLR before surgery or before the beginning of systemic treatments, limiting the evaluation of their prognostic role only at a pre-treatment phase. However, chemotherapy and especially tumor burden can influence chronic inflammation and therefore can have an impact of the lymphocyte ratios. Conversely, disease progression or changes in the molecular phenotype of cancer, can be reflected by an alteration in NLR, MLR and PLR.

Our data showed that variation of these ratios is associated with worse prognosis, in particular when an increase is detected. When considering tumor burden, only NLR was found to be associated with an increase in metastatic sites (p = 0.04). However, it is important to notice that the number of metastatic sites might not reflect the real tumor burden and that progression may also occur without an increase in the number of sites. Therefore, further evaluations are required in order to understand the relationship between MLR, NLR, PLR, tumor burden and progression.

Although exploratory, the present study gives distinct insights that could have several immediate and future clinical applications. These results offer biologically driven LR cut-offs that could be used not only for patients’ stratification and clinical trial design but also for clinical monitoring, since longitudinal variations in LRs are informative of the disease evolution. Moreover, as immunotherapy is gradually gaining momentum also in TNBC, cost-effective immunity-driven biomarkers could have an interesting role as observed in other cancer types^51–53^.

Notably, exosomes are capable to influence the immune system by acting on monocyte maturation, therefore posing some intriguing perspectives on the integration of LRs with the exosomal characterization^28^. If on one hand these results pose the basis for a potential monitoring tool capable to highlight sites that could be more prone to distant involvement, on the other, the study confirms the interaction between the metastatic niche and the immune system and offers an interesting rationale for targeting such interactions for future treatment strategies.

## Conclusions

The present study was able to demonstrate that elevated monocyte-to-lymphocyte and neutrophil-to-lymphocyte ratios are significantly associated with cancer, as they present lower values in healthy subjects. These ratios significantly vary among different breast cancer subtypes, reflecting the different involvement of the immune system in HER2-positive and triple negative disease. Furthermore, monocyte-to-lymphocyte and neutrophil-to-lymphocyte ratios are confirmed as prognostic factors and the proposed score suggested their possible combination in order to stratify patients younger than 65 years old in three different risk subgroups that consider aspects linked both to systemic inflammation and immunodepression.

Interestingly, the reported findings suggested an important interplay between immunity, specific circulating exosomal subpopulations and metastatic sites, suggesting the possibility to infer information on the organ-specific metastatic process.

Albeit the purpose of the study was exploratory, these data suggest that further investigations should be considered on this topic to better dissect immunoediting and homing mechanisms of circulating tumor cells.

## Supplementary information

Supplemental figures.
