# Supplementary material for: Biologically driven cut-off definition of lymphocyte ratios in metastatic breast cancer and association with exosomal subpopulations and prognosis
Source: Sci Rep. 2020 Apr 24;10:7010. doi: 10.1038/s41598-020-63291-2 (PMC7181663; doi:10.1038/s41598-020-63291-2)
Supplement: Supplementary file 1 — Supplemental figures. [file 41598_2020_63291_MOESM1_ESM.pdf]

Biologically driven cut-off definition of lymphocyte ratios in metastatic breast cancer and association with exosomal subpopulations and prognosis.

Gerratana L[1,2,3*#], Basile D[1,3#], Toffoletto B[4], Bulfoni M[4], Zago S[5], Magini A[6], Lera M[1], Pelizzari G[1,3], Parisse P[7,8], Casalis L[8], Vitale MG[1,3], Fanotto V[1,3], Bonotto M[3], Caponnetto F[1], Bartoletti M[1,3], Lisanti C[1,3], Minisini AM[3], Emiliani C[6], Di Loreto C[1,4], Fasola G[3], Curcio F[1,5], Beltrami AP[1,4], Cesselli D[1,4§], Puglisi F[1,9§]

[#] Contributed equally [§] Contributed equally

1. Department of Medicine (DAME), University of Udine, Udine, 33100, Italy
2. Department of Medicine, Division of Hematology and Oncology, Robert H Lurie Comprehensive Cancer Center, Feinberg School of Medicine, Northwestern University, Chicago, IL 60611, USA
3. Department of Oncology, ASUFC University Hospital, Udine, 33100, Italy
4. Anatomic Pathology Institute, ASUFC University Hospital, Udine, 33100, Italy
5. Clinical Pathology Institute, ASUFC University Hospital, Udine, 33100, Italy
6. Department of Chemistry, Biology and Biotechnology, University of Perugia, Perugia, 06122, Italy
7. INSTM-ST Unit, Area Science Park, Trieste, 34149, Italy
8. Elettra-Sincrotrone Trieste S.C.p.A., Area Science Park, Trieste, 34149, Italy
9. Department of Medical Oncology, Centro di Riferimento Oncologico (CRO), IRCCS, 33081, Aviano, Italy

*** Correspondence to:**

Lorenzo Gerratana, MD

Department of Medicine (DAME), University of Udine, Piazzale Kolbe 4, Udine, 33100, Italy

email: gerratana.lorenzo@spes.uniud.it

Figure S1: Different MLR (A), NLR (B) and PLR (C) distribution according to molecular profile in respect to healthy subjects. The red line indicates the median value of the single lymphocyte ratio among donors.

Figure S2: Kaplan Meier plot in terms of OS according to NLR-MLR Score. P value was calculated though log-rank test.

Figure S3: Original version of Figure 1G immunoblot

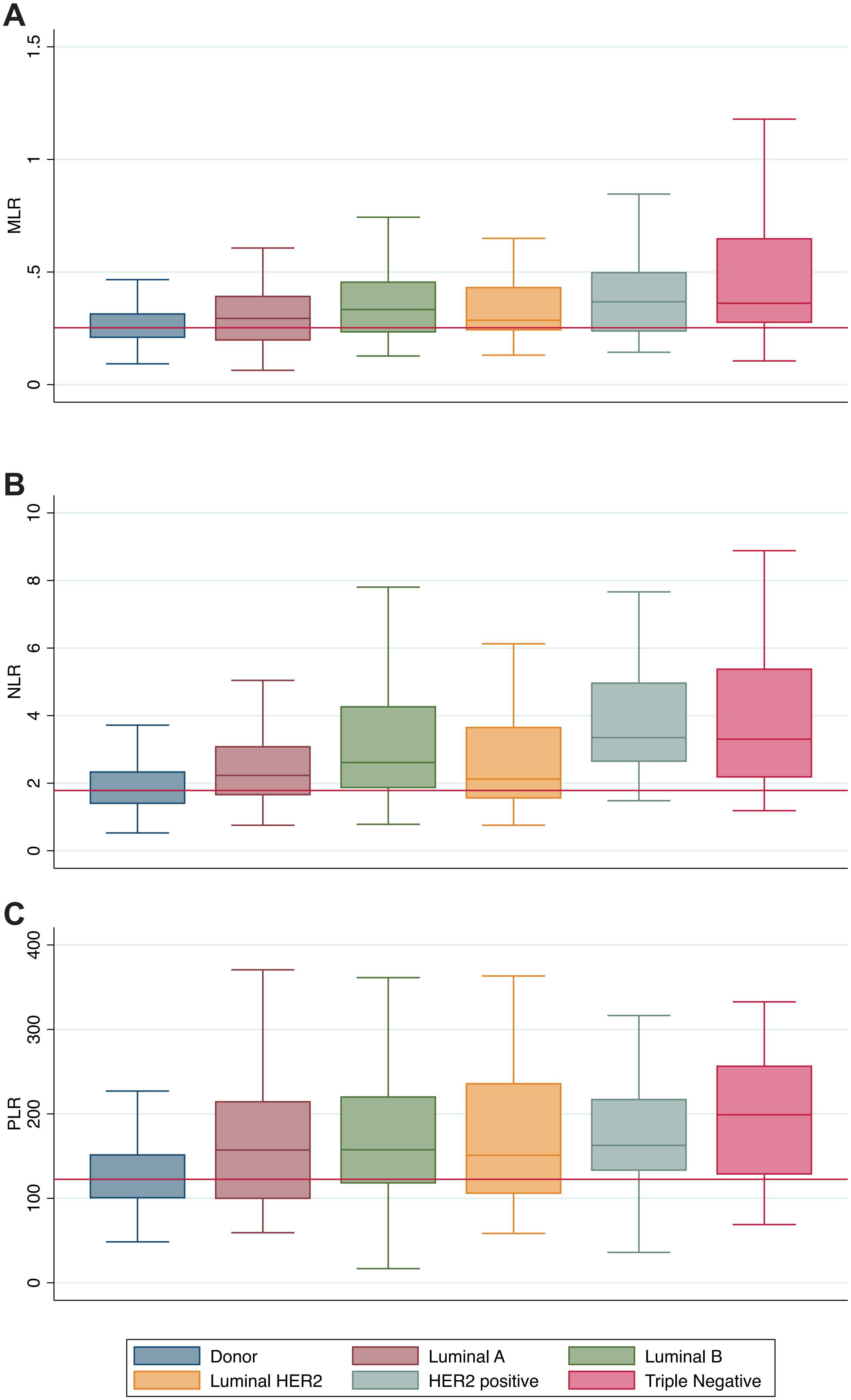
A
MLR
0
.5
1
1.5
B
NLR
0
2
4
6
8
10
C
PLR
0
100
200
300
400
Donor
Luminal A
Luminal B
Luminal HER2
HER2 positive
Triple Negative

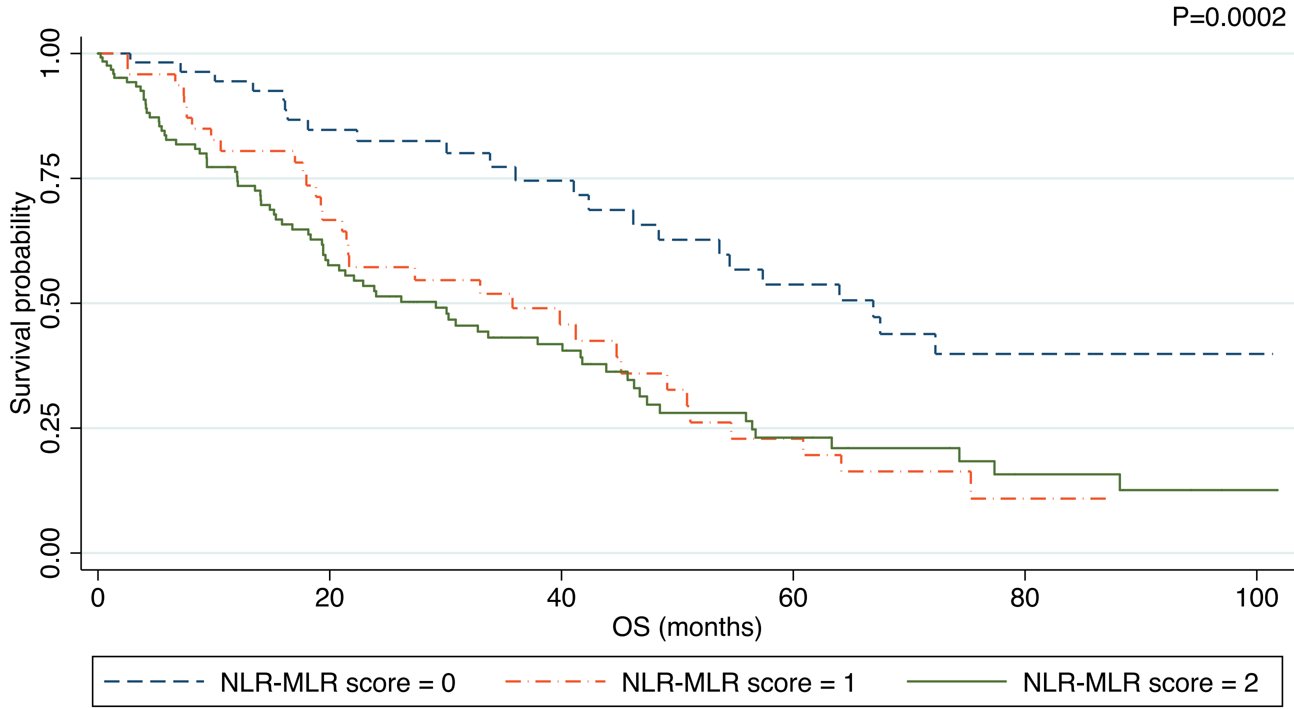
P=0.0002
Survival probability
1.00
0.75
0.50
0.25
0.00
0
20
40
60
80
100
OS (months)
NLR-MLR score = 0
NLR-MLR score = 1
NLR-MLR score = 2

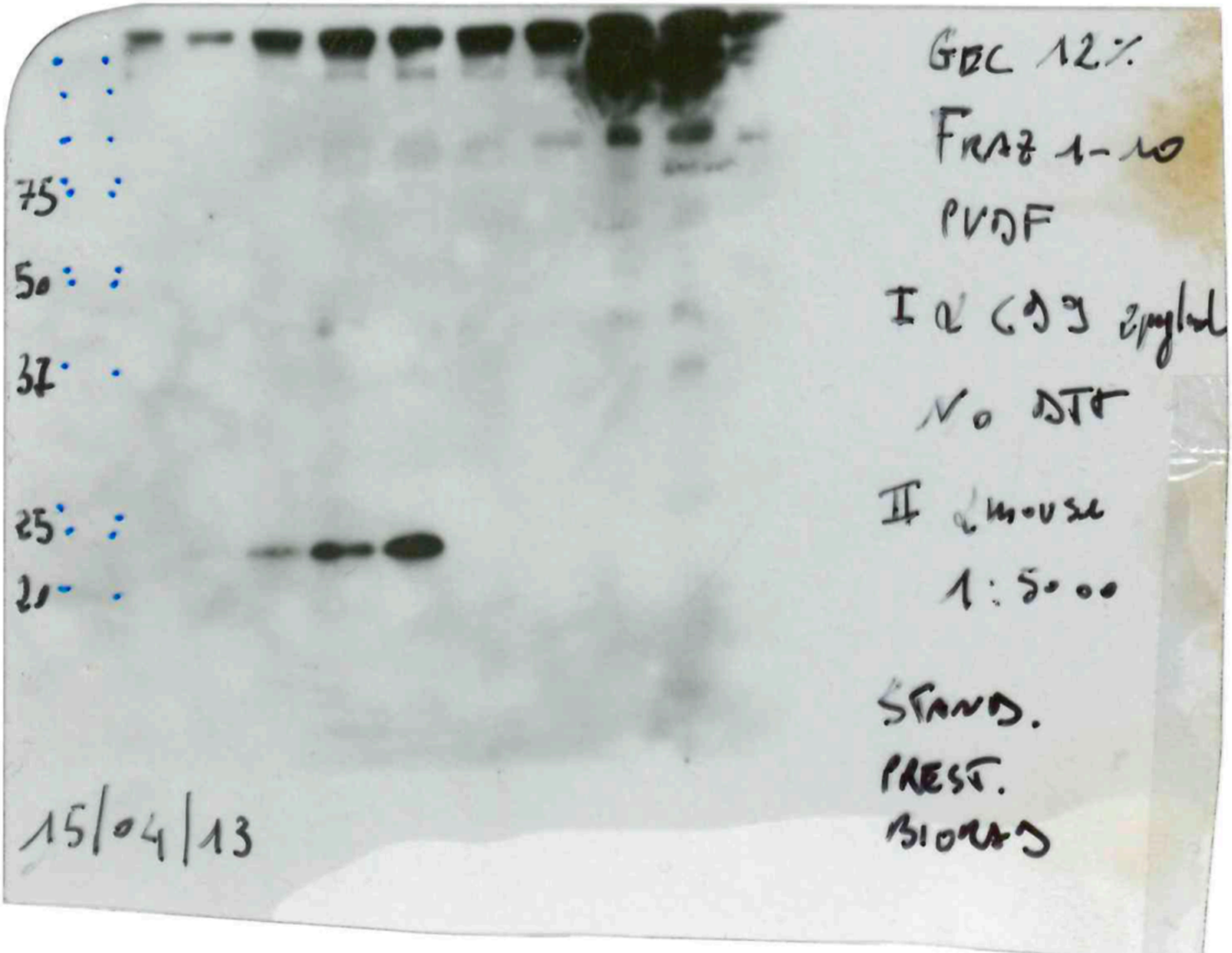
75
50
37
25
GEL 12%
Fraz 1-10
PVDF
No DTT
II
mouse
1:5000
STAND.
PREST.
BIORAD
15/04/13
